# Emergent Re‐Do Open Aortic Valvular Repair and Foreign Body Retrieval During TAVR: A Case Report

**DOI:** 10.1155/cria/1207682

**Published:** 2026-11-01

**Authors:** Ryan Dougherty, Thida Ong, Michael Morgan, Eric Chiang, Aariya Srinivasan, Sabry Ayad

**Affiliations:** ^1^ Department of Anesthesiology Research Integrated Hospital Institute Cleveland Clinic Foundation Cleveland Ohio USA clevelandclinic.org; ^2^ Department of Anesthesiology Outcomes Research Consortium Houston Texas USA or.org

**Keywords:** aortic stenosis, cardiac arrest, cardiopulmonary bypass, TAVR

## Abstract

A 73‐year‐old man presented for a transcatheter aortic valve replacement after prosthetic aortic valve dysfunction. During the case, the transvenous pacer lost capture, causing the new prosthetic valve to embolize to the ascending aorta. This case details the intraoperative management throughout the valve deployment, attempted retrieval, and subsequent emergent redo open aortic valve repair with foreign body removal. Complications and considerations of a redo open chest, vasoplegia, cardiopulmonary bypass, aortic dissection, right ventricular failure, heart block, and prosthetic valve embolization are discussed.

## 1. Case History and Examination

The patient is a 73‐year‐old male who presented to a large academic tertiary care hospital with significant experience in both cardiac procedures and surgery for elective transcatheter aortic valve replacement (TAVR) secondary to prosthetic aortic valve dysfunction. The patient has a past medical history of hypertension, severe aortic stenosis, aortic valve regurgitation, ascending thoracic aortic aneurysm status postascending aortic graft with aortic valve replacement, New York Heart Association Class II heart failure with preserved ejection fraction, mildly dilated right ventricle (RV), and moderate to severe pulmonary hypertension. The patient began having a persistent cough with exertional dyspnea, and it was determined that the prosthetic aortic valve had failed. Cardiac surgery and interventional cardiology were consulted. It was recommended that he undergo a valve‐in‐valve TAVR. During prosthetic valve deployment, the transvenous pacemaker lost capture, resulting in loss of rapid ventricular pacing. This caused the new prosthetic valve to embolize into the ascending aorta, necessitating emergent cardiopulmonary bypass (CPB) surgery.

### 1.1. Preoperative Investigations and Diagnosis

Computed tomography angiography 2 months prior to elective TAVR reported minimal calcification of the bioprosthetic aortic valve. Prolapse of the left prosthetic valve was seen. The aortic root was dilated to 4.2 cm with mild calcifications of the native wall segments.

ECHO 1 month prior to surgery was performed. Both the left and right atrial cavities were severely dilated. Mild posterior left ventricular (LV) hypertrophy with ejection fraction (LVEF) of 55% without any wall motion abnormalities was noted. The RV displayed mild dilation and decreased systolic function with estimated systolic pressures of 65 mmHg. The right atrial pressure was estimated at 15 mmHg. Diastolic function evaluation of the LV was not performed due to severe aortic insufficiency. The mean velocity across the aortic valve was 193 cm/s, and the dimensionless valve index was 0.37. Other valvular dysfunctions included 1–2+ mitral regurgitation, 1–2+ tricuspid regurgitation, 3+ aortic valve regurgitation, and 1+ pulmonic valve regurgitation.

## 2. Management

The patient was placed under monitored anesthesia care with 0.5 mcg/kg dexmedetomidine IV bolus followed by 0.5 mcg/kg/hr dexmedetomidine IV infusion. Vital signs immediately prior to induction were HR 84 beats per minute (bpm) with premature ventricular complexes, blood pressure (BP) 183/64 mmHg, and pulse oxygen saturation (SpO2) 100% on 8 L simple mask. IV dexmedetomidine was subsequently titrated between 0.2 and 0.6 mcg/kg/hr based on patient responsiveness, HR, and BP. Invasive arterial access and monitoring were obtained following sedation by interventional cardiology. An 8‐French sheath was placed by interventional cardiology in the right femoral artery followed by a 5‐French sheath inferior to the first sheath. In addition, a 5/6‐French arterial sheath was placed in the right radial artery through which a sentinel embolic capture device was placed into the innominate and left carotid arteries. A transvenous pacing wire was introduced through the right femoral vein and placed into the RV under fluoroscopy by interventional cardiology. Transvenous pacing was tested, and capture was obtained at 1.00 mA. The patient was paced at 180 bpm during valve deployment. However, during this period, the pacemaker lost capture, causing return of native function and the new prosthesis to embolize the ascending aorta. The delivery balloon was left inflated to reposition the prosthesis back to the descending aorta. While this was initially successful, the valve repeatedly re‐embolized to the ascending aorta with deflation of the balloon. The prosthesis was able to be moved from the large ostium of the innominate to the left subclavian after exchanging the initial 14 French wire for a 16 French. Again, following deflation of the balloon, the prosthesis re‐embolized to the innominate artery. Following discussion between interventional cardiology, vascular surgery, and cardiothoracic surgery, a subsequent attempt was made to anchor the distal end of the prosthesis to the aorta proximal to the innominate artery using a Palmaz stent (Cordis). The patient was paced at 180 bpm while the stent was deployed. After multiple unsuccessful attempts at pacing, the patient developed *R* on *T* ventricular fibrillation. Chest compressions and bag‐mask ventilation were initiated, and the patient was defibrillated. Postdefibrillation vital signs were HR 73 bpm, BP 115/18 mmHg on arterial line, and 88% SpO2 on bag‐mask. The decision was made to intubate the patient at this time. He was given an 80 mg propofol IV bolus in a split dose and 100 mg rocuronium IV. The patient was intubated with a 7.5‐mm endotracheal tube and McGrath (Medtronic) video laryngoscope. The patient was briefly started on 1% isoflurane, resulting in a MAC of 0.7. Dexmedetomidine infusion was weaned. A phenylephrine IV infusion was started and titrated up to a maximal dose of 90 mcg/min. Subsequent attempts were made at retrieving the embolized prosthesis percutaneously with new balloons, but these were unsuccessful. With the prosthesis secured to the ascending aorta proximal to the innominate, by the wire and balloon, the decision was made to cease percutaneous retrieval attempts. Isoflurane was stopped, and a 30‐mcg/kg/min propofol IV infusion was started for transport. The patient was taken emergently for a re‐do open aortic valve repair with foreign body retrieval.

The patient was transported, intubated, ventilated, and sedated from the TAVR suite to an OR capable of CPB. Vital signs on arrival were HR 60 bpm, BP 141/38 mmHg, and 100% SpO2 on 90% FiO2. The propofol infusion was discontinued, and anesthesia was maintained with 0.6% isoflurane. A 9‐French double lumen central line was placed in the right internal jugular vein under ultrasound guidance. Following placement of the central line, a 7.5‐French pulmonary artery catheter was introduced through one of the central line ports.

Intraoperative transesophageal ECHO (TEE) (Figure 1) following new prosthetic aortic valve embolization was performed. TEE reported LV and RV dilation and an LVEF of 40%. A moderate decrease in RV systolic function was noted, along with a venous catheter in the RV. 1+ mitral valve regurgitation and 2+ tricuspid valve regurgitation were noted. Severe, 4+, aortic valve regurgitation with regurgitant jets at the right and noncoronary cusps was observed, most likely due to previous prosthetic valve dysfunction or tear.

**FIGURE 1:**
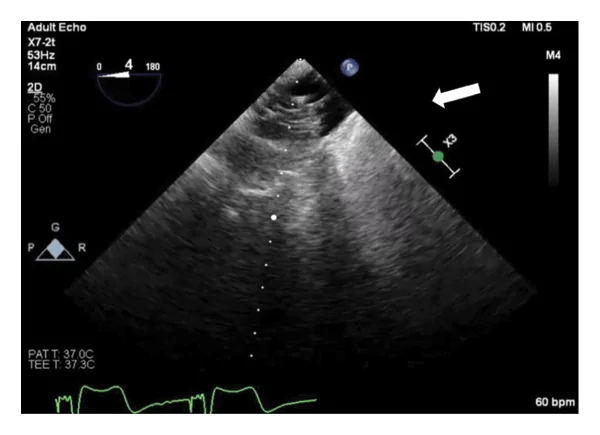
Intraoperative transthoracic echocardiography of the aortic arch in long axis view displaying embolized prosthetic valve.

The patient was given 40,000 units of heparin in a split dose for anticoagulation prior to initiating CPB. Arterial blood gas (ABG) analysis obtained immediately prior to starting bypass reported a pH of 7.31, base excess of −5, lactate of 2.1, Hgb of 9.9 g/dL, and an activated clotting time (ACT) of 486 s. Vital signs prior to starting CPB were HR 60 bpm, BP 108/41 mmHg, mean arterial pressure (MAP) 66, pulmonary artery pressure (PAP) 49/30 mmHg, mean PAP (MPAP) 35 mmHg, and central venous pressure (CVP) 18 mmHg. Aortic cannulation was performed by cardiac surgery through the previous aortic prosthetic graft. A cannula was also placed in the superior vena cava for retrograde cerebral perfusion (RCP). CPB was started, and the patient was actively cooled to 20°C through ice on his head and in his chest. ACTs were regularly checked and maintained above 500 s while on CPB. At 20°C, the CPB pump was stopped, hypothermic circulatory arrest was achieved, and RCP was started. An incision was made below the innominate artery in the proximal aortic arch. The embolized prosthetic valve and securing devices were removed through this incision. The patient was subsequently rewarmed to 32°C, and circulatory arrest was stopped 22 min after initiation. ABG immediately following circulatory arrest reported a pH of 7.28, base excess of −6, lactate of 3.4 mmol/L, and Hgb of 7.7 g/dL. A cross clamp was placed on the previous aortic prosthetic graft. Anterograde and retrograde cold cardioplegic solutions were introduced to obtain cardiac immobility. An aortotomy was made, and the old prosthetic valve was gently removed. A new 23‐mm prosthesis was sutured into place, and the incisions were closed. The aortic cross clamp was in place for 2 h. During this time, ABG indicated a decrease in Hgb to 8.8 g/dL. As the patient was weaned from CPB, he was started on IV infusion of 0.375 mcg/kg/minute milrinone, 4 mcg/minute of norepinephrine, 0.04 units/minute of vasopressin, and 4 mcg/min of epinephrine. Two right atrial and RV epicardial wires were placed for sinus bradycardia with first‐degree atrioventricular block and intermittent junctional bradycardia. Vital signs immediately following CPB cessation were HR 84 bpm with epicardial pacing, BP 89/54 mmHg, MAP 64, PAP 38/20 mmHg, MPAP 24, and CVP 8 mmHg. Following CPB cessation, vasopressors were titrated to maximal doses of 0.08 units/minute of vasopressin, 18 mcg/min of norepinephrine, and 8 mcg/min of epinephrine. Heparin was reversed with 250 mg protamine IV. ABG and ACT after CPB stopped indicated a pH of 7.22, lactate of 6.8 mmol/L, and a clotting time of 170 s. An additional 50 mg of protamine IV was given.

Throughout both procedures, the patient received a total of 7 units of pRBC, 6 units of platelets, 8 units of FFP, 4 units of cryoprecipitate, 3.1 L of Cell Saver (Haemonetics Corporation), 500 mL of 5% albumin, 2.8 L of lactate Ringer′s, 700 mL of normal saline, and 1.7 L of Plasma‐Lyte (Baxter International).

Intraoperative TEE at the conclusion of the procedure reported moderately reduced LV and RV function with dilation, improved tricuspid regurgitation, and no evidence of aortic dissection following decannulation.

The patient was transported to the surgical ICU, intubated, ventilated, sedated on 25 mcg/kg/min IV propofol, and on the vasopressor support regimen mentioned above. Throughout the procedure, bispectral index (BIS) monitoring was utilized. The average BIS score was 37 during the procedure except for the 22 min of circulatory arrest, during which the BIS reading was zero. Due to concerns of encephalopathy following surgery, the patient’s temperature was maintained at < 36°C for 48 h following admission to the ICU. Bedside EEG was performed and demonstrated severe diffuse encephalopathy. EEG did not report epileptic or seizure activity. The encephalopathy improved on Postoperative Day 2. He was subsequently weaned from vasopressors and extubated.

### 2.1. Outcome and Follow‐Up

On Postoperative Day 3, the patient began complaining of back pain. Due to concern for a small intimal tear of the aorta and increasing BP, the patient was started on a nitroprusside IV infusion. This was titrated up to a maximum dose of 80 mcg/min with the highest recorded BP of 145/56 mmHg. Computed tomography angiography was unable to be completed during the patient’s hospital course due to persistently elevated creatinine starting on Postoperative day 1 and concerns of worsening kidney function with administration of IV contrast dye. Nitroprusside infusion was weaned on Postoperative Day 5 as his BP improved.

Electrophysiology was consulted for complete heart block and ongoing atrioventricular pacing requirement. During his ICU course, the patient developed a rhythm of atrial fibrillation with fascicular escape underlying the epicardial paced rhythm. A 150‐mg bolus of IV amiodarone was given, followed by a 0.5 mg/min infusion for chemical rhythm conversion of atrial fibrillation. On Postoperative Day 5, the patient underwent placement of a dual‐chamber permanent pacemaker under procedural sedation. A Medtronic Azure XT DR MRI Surescan was placed and programmed to DDDR.

Oral amiodarone replaced the IV formulation as the patient transferred from the ICU. Elevated liver enzymes and jaundice secondary to amiodarone necessitated tapering. The patient remained in paced sinus rhythm as amiodarone dosage decreased.

Nitroprusside infusion was continued for systolic BP greater than 130 mmHg until Postoperative Day 5. Amlodipine was increased from a prior‐to‐admission dose of 5 mg–10 mg daily. Nitroprusside was subsequently tapered. The patient was then transferred out of the ICU on Postoperative Day 6.

## 3. Discussion

### 3.1. Redo Chest

Initial open chest surgeries are associated with a risk of mortality as low as 5%. Repeat open chest procedures, however, are associated with a mortality risk as high as 19%. Specifically, repeat aortic root replacement has been associated with a mortality risk of 12%–14% even in the most skilled facilities. Despite these risks, the number of redo aortic cases has quickly trended upward since 1998 [1]. Multiple reasons for this increased risk have been noted, including previous surgical scarring, increased age at time of redo, combined procedures, peripheral arterial disease, coronary artery disease, history of myocardial infarction, lung disease, mitral valve replacement, coronary artery bypass grafting, and emergency procedures [2, 3].

### 3.2. CPB‐Associated Vasoplegia

In a review of current guidelines, Ortoleva et al. defined vasoplegia as a decreased MAP and systemic vascular resistance (SVR) with a normal or increased cardiac index (CI). There is not, however, a strict definition of vasoplegia that details at what value vasoplegia occurs. Vasoplegia is thought to be due to a combination of endothelial dysfunction, nitric oxide deregulation, vasopressin deficiency, and prostaglandin release. Vasoplegia‐related CPB specifically refers to vasoplegia that is resistant to vasopressor support. While it is difficult to characterize the overall prevalence of vasoplegia due to varying definitions, the prevalence of CPB vasoplegia in patients undergoing cardiac surgery is estimated at 20%. Specifically, in patients undergoing aortic valve surgery, CPB vasoplegia may be seen in as high as 10% of patients. Vasoplegia may be seen at initiation of CPB through a patient’s time in the ICU. Of the patients who encounter vasoplegia during CPB, there is an associated increase in length of stay in both the ICU and hospital, an increase in kidney injury, and an increase in mortality. Treatment of vasoplegia during CPB begins with optimization of the CPB circuit by adjusting the flow rate and verifying monitor accuracy. Intravascular pathologies such as aortic dissection must also be ruled out in the setting of sudden vasoplegia. Vasopressor support should be considered in patients who fail initial optimization. Traditional vasopressors such as phenylephrine, norepinephrine, epinephrine, and vasopressin should be considered as first‐line pharmacologic treatment. In at least two trials, initiation of vasopressin as a first‐line vasopressor led to significantly lower need for hemodynamic support postbypass. In those patients who do not respond to traditional vasopressors, off‐label use of medications such as methylene blue, hydroxocobalamin, prostaglandin inhibitors, and Angiotensin II may be considered. While hydroxocobalamin is a comparative newcomer for the treatment of vasoplegia, methylene blue has been used for refractory hypotension for nearly 20 years. Hydroxocobalamin directly inhibits both nitric oxide and nitric oxide synthase. Methylene blue, on the other hand, only inhibits nitric oxide synthase, thereby limiting nitric oxide production. Other off‐label therapies for vasoplegia that have shown mixed success include prostaglandin inhibitors, Angiotensin II, hydroxocobalamin, and a hydrocortisone–ascorbic acid–thiamine combination. In addition, corticosteroids have been suggested to be of benefit in vasodilatory shock in the ICU; however, they may be potentially less useful in the acute phase of vasoplegia [4].

### 3.3. Aortic Dissection

Intraoperative aortic dissection is a rare but known risk of any cardiac surgery. Williams et al. found the prevalence of aortic dissection related to surgery was 0.04% for patients undergoing coronary artery bypass grafting on CPB and 0.06% for those without CPB. Despite occurring intraoperatively, with full operating room resources available, there is still a 48% mortality. Multiple causes have been proposed, including CPB cannulation technique, aortic cross‐clamping, and aortic balloon pump; however, an exact mechanism has not been detailed. In patients who experience intraoperative aortic dissection, there will be brisk expansion of the aorta and significant bleeding surrounding the surgical site. CPB should be stopped as soon as possible, and cannulation sites moved until the defect can be repaired [5].

TAVR procedure is a known risk factor for iatrogenic aortic dissection. As shown in Figure 2, approximately 0.1%–1.9% of patients who underwent TAVR resulted in aortic dissection. While this is a rare complication, mortality rates can be as high as 52% despite immediate access to diagnosis and treatment [6]. In a small single‐center retrospective study conducted in Japan, aortic dissection was hypothesized to be due to valve implantation and direct injury by the edge of the prosthetic heart valve, the catheter, and presurgical dilatation [7]. A single‐center trial, conducted at the University of Pittsburgh, supports some of these risk factors. In the Pittsburgh study, 44% of Type A dissections were caused by posterior annular rupture by the prosthetic valve, 22.2% were caused by displacement of the prosthetic valve, and 11.1% of Type A dissections were the result of embolization of the prosthetic valve [8]. In addition, self‐expanding valves have been noted to carry an increased risk of postoperative dissection compared to balloon‐inflatable devices. Type A dissections are commonly diagnosed by TEE following deployment of the prosthesis, but some dissections may not appear until multiple days after surgery. Standard treatment for a Type A dissection is an emergent open surgical repair. Patients who are poor open surgical candidates may be considered for endovascular repair assuming they have space to accommodate a stent without stretching the aortic valve, occluding the brachiocephalic artery, or occluding previous grafts [6].

**FIGURE 2:**
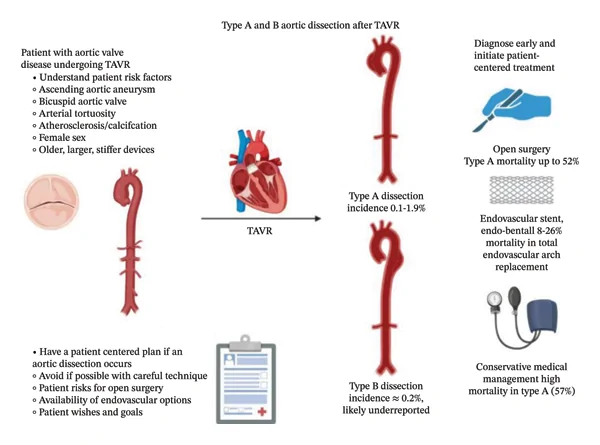
Types, incidence, and management of aortic dissections. Adapted from aortic dissection following transcatheter aortic valve replacement, DeGraaf, et al. [6].

### 3.4. Heart Block and Permanent Pacemaker Placement

According to Sammour et al., high‐grade atrioventricular block and novel left bundle branch block (LBBB) are the most common conduction abnormalities immediately following TAVR. LBBB is most commonly seen intraoperatively and up to 24 h postoperative; however, up to 30% of patients may not display symptoms of AV block until Postoperative Day 2. Preexisting conduction abnormalities, surgical manipulation, brand and model of replacement valve, diabetes, female sex, and a history of coronary bypass graft are all factors influencing risk of new postoperative AV block and LBBB. New‐onset LBBB has been shown in multiple trials to have a significant increase in mortality and pacemaker placement up to 1 year after TAVR. Despite this, up to half of patients with acute‐onset high‐grade AV blockade may quickly self‐resolve regardless of pacemaker placement [9].

### 3.5. Prosthetic Valve Embolization

Prosthetic valve embolization and valve inversion are known but rare complications during TAVR. Embolization during TAVR is estimated to occur in 0.92% of cases, with the embolized prosthetic valve most commonly moving to the ascending aorta [10]. Predisposing factors to valve embolization have been suggested to be caused by placement too proximal to the aorta, placement of the prosthetic valve out of alignment compared to the native valve’s orientation, sinus of Valsalva size, sinotubular junction size, ascending aorta size, presence of a bicuspid aortic valve, prosthesis type and generation, ability to re‐sheath the prosthesis, procedural time, fluoroscopy time, and volume of contrast used [10, 11]. Issues with rapid ventricular pacing may be harder to specifically identify. The Transcatheter Heart Valve Embolization and Migration (TRAVEL) registry analyzed rapid pacing failure on a case‐by‐case basis due to an array of factors; however, they estimated that rapid ventricular pacer failure occurred in 4.8% of the cases examined [10].

### 3.6. Rapid RV Pacing

Rapid RV pacing is used in TAVR to create a stable procedural field for prosthetic deployment and manipulation. The HR is artificially increased to a point where the cardiac output temporarily decreases. However, despite this vital role in the TAVR procedure, there is a dearth of guidelines on how to safely induce and maintain RV rapid pacing. Newer methods such as LV pacing have shown promising results and pacing failure rates as low as 1% [12].

### 3.7. Retrieval of Embolized Prosthetic Valve

Management of an embolized prosthetic valve depends on patient’s hemodynamic stability. In a hemodynamically stable patient, percutaneous retrieval attempts may be considered. According to the TRAVEL registry, percutaneous reposition attempts were made in 41% of cases with a 51.8% success rate. Only 19% of cases seen in the TRAVEL registry required surgical intervention, and of those, 21.6% required CPB [10]. In percutaneous attempts, a snare was most commonly used; however, balloons and self‐expanding stents have shown to be useful in prosthesis retrieval [10, 11, 13]. In at least one case, the prosthetic valve was successfully trapped against the vessel wall by use of two additional valves placed on the leaflets of the embolized prosthetic valve [14]. In the case of prosthetic inversion, keeping a guidewire in place through the valve orifice during adjustment has been suggested to maintain correct orientation and prevent inversion [11].

## 4. Conclusion

Transcatheter aortic valve repair has become a common procedure in place of open cardiac surgery. Complication rates are low; however, when they do occur, there are significant mortality implications. Embolization of the prosthetic valve is a rare complication with complex management dependent upon the stability of the patient. In addition, management of an embolized prosthetic valve carries its own risk of damage and complications. Expedited multidisciplinary management between anesthesia, cardiac surgery, interventional cardiology, and intensive care teams is critical to successful retrieval and stabilization of an embolized prosthetic valve.

## Funding

No funding was received for this study.

## Ethics Statement

This article is a retrospective look at a single patient’s care. It has not been reviewed by an ethics committee, and due to the type of study, it is not required by institutional guidelines.

## Consent

An explanation of the case report above, including patient information utilized, educational intent, and intended publication location, was provided to the patient. Written consent for chart review and publication of nonidentifying information was obtained prior to publication.

## Conflicts of Interest

The authors declare no conflicts of interest.

## Practical Application

This is a retrospective evaluation of a single case; therefore, generalizability is limited.

## Supporting Information

Additional supporting information can be found online in the Supporting Information section.

## Supporting information


**Supporting Information** CARE checklist.

## Data Availability

The data that support the findings of this study are available on request from the corresponding author. The data are not publicly available due to privacy or ethical restrictions.
