# Supplementary material for: Emergent Re‐Do Open Aortic Valvular Repair and Foreign Body Retrieval During TAVR: A Case Report
Source: Case Rep Anesthesiol. 2026 Nov 1;2026:1207682. doi: 10.1155/cria/1207682 (PMC13725070; doi:10.1155/cria/1207682)
Supplement: Supplementary file 1 [file CRIA-2026-1207682-s001.pdf]

CARE case report guidelines

CARE Checklist of information to include when writing a case report

(cc) BY-NC-ND

Topic	Item	Checklist item description	Reported on Line
Title	1	The diagnosis or intervention of primary focus followed by the words "case report"	1
Key Words	2	2 to 5 key words that identify diagnoses or interventions in this case report, including "case report"	7
Abstract (no references)	3a	Introduction: What is unique about this case and what does it add to the scientific literature?	10
	3b	Main symptoms and/or important clinical findings	14
	3c	The main diagnoses, therapeutic interventions, and outcomes	12
	3d	Conclusion—What is the main "take-away" lesson(s) from this case?	15
Introduction	4	One or two paragraphs summarizing why this case is unique (**may include references**)	19-32
Patient Information	5a	De-identified patient specific information.	34-48
	5b	Primary concerns and symptoms of the patient.	64-66
	5c	Medical, family, and psycho-social history including relevant genetic information	19-48
	5d	Relevant past interventions with outcomes	35-48
Clinical Findings	6	Describe significant physical examination (PE) and important clinical findings.	63-89
Timeline	7	Historical and current information from this episode of care organized as a timeline	51-174
Diagnostic Assessment	8a	Diagnostic testing (such as PE, laboratory testing, imaging, surveys).	97-103
	8b	Diagnostic challenges (such as access to testing, financial, or cultural)	N/a
	8c	Diagnosis (including other diagnoses considered)	66, 108-121
	8d	Prognosis (such as staging in oncology) where applicable	n/a
Therapeutic Intervention	9a	Types of therapeutic intervention (such as pharmacologic, surgical, preventive, self-care)	67-81; 127-165
	9b	Administration of therapeutic intervention (such as dosage, strength, duration)	76-89; 127-165
	9c	Changes in therapeutic intervention (with rationale)	69-89
Follow-up and Outcomes	10a	Clinician and patient-assessed outcomes (if available)	176-198
	10b	Important follow-up diagnostic and other test results	176-198
	10c	Intervention adherence and tolerability (How was this assessed?)	75-89
	10d	Adverse and unanticipated events	64-87
Discussion	11a	A scientific discussion of the strengths AND limitations associated with this case report	337-338
	11b	Discussion of the relevant medical literature **with references**.	201-326
	11c	The scientific rationale for any conclusions (including assessment of possible causes)	N/a
	11d	The primary "take-away" lessons of this case report (without references) in a one paragraph conclusion	328-335
Patient Perspective	12	The patient should share their perspective in one to two paragraphs on the treatment(s) they received	N/a
Informed Consent	13	Did the patient give informed consent? Please provide if requested	Yes ☑ No ☐
